# Acceptance, Use, and Barriers of Telemedicine in Transgender Health Care in Times of SARS-CoV-2: Nationwide Cross-sectional Survey

**DOI:** 10.2196/30278

**Published:** 2021-12-03

**Authors:** Stefan Hertling, Doreen Hertling, David Martin, Isabel Graul

**Affiliations:** 1 Department of Obstetrics and Gynaecology University Hospital Jena Jena Germany; 2 Orthopaedic Department Campus Eisenberg University Hospital Jena Eisenberg Germany; 3 Department of Human Medicine Faculty of Health Witten/Herdecke University Witten Germany; 4 Department of Gynaecology Hospital Rummelsberg University Hospital Erlangen Rummelsberg Germany; 5 Department of Trauma, Hand and Reconstructive Surgery University Hospital Jena Jena Germany; 6 Orthopaedic Department Universitätsklinikum Halle Halle Germany

**Keywords:** telemedicine, health services research, COVID-19, transgender health care, acceptance, usage, barrier, telehealth, gender, identity, transgender, cross-sectional, survey, Germany, framework, implementation

## Abstract

**Background:**

The global incidence in the treatment of transgender people is increasing. During the COVID-19 pandemic, many consultations had to be cancelled, postponed, or converted to a virtual format. Telemedicine in the management of transgender health care could support physicians.

**Objective:**

The aim of this study was to analyze the acceptance, use, and barriers of telemedicine in transgender health care in times of SARS-CoV-2 in Germany.

**Methods:**

This prospective cross-sectional study was based on a survey of gynecological endocrinologists and transgender patients undergoing gender-affirming hormone treatment in Germany during the COVID-19 pandemic. Descriptive statistics were calculated, and regression analyses were performed to show correlations.

**Results:**

We analyzed responses of 269 transgender patients and 202 gynecological endocrinologists treating transgender patients. Most believed that telemedicine was useful. Physicians and patients rated their knowledge of telemedicine as unsatisfactory. The majority of respondents said they did not currently use telemedicine, although they would like to do so. Patients and physicians reported that their attitudes toward telemedicine had changed positively and that their use of telemedicine had increased due to COVID-19. The majority in both groups agreed on the implementation of virtual visits in the context of stable disease conditions. In the treatment phases, 74.3% (150/202) of the physicians said they would use telemedicine during follow-ups. Half of the physicians said they would choose tele-counseling as a specific approach to improving care (128/202, 63.4%). Obstacles to the introduction of telemedicine among physicians included the purchase of technical equipment (132/202, 65.3%), administration (124/202, 61.4%), and poor reimbursement (106/202, 52.5%).

**Conclusions:**

Telemedicine in transgender health care found limited use but high acceptance among doctors and patients alike. The absence of a structured framework is an obstacle for effective implementation. Training courses should be introduced to improve the limited knowledge of physicians in the use of telemedicine. More research in tele-endogynecology is needed. Future studies should include large-scale randomized controlled trials, economic analyses, and the exploration of user preferences.

## Introduction

The global incidence in the treatment of transgender people is increasing. Approximately 15,000 to 25,000 persons in Germany are affected [1]. According to the World Health Organization, transgenderism refers to people whose perceived gender and the physical gender they were assigned at birth do not coincide [2]. As a socially and medically vulnerable group, transgender people face numerous inequalities in terms of health and mental health problems as compared to cisgender people [3]. The COVID-19 pandemic has exacerbated international health problems and is creating devastating mental health strains on a global scale for many populations. Transgender people today face problems related to social, physical, and mental well-being, as well as difficulties in accessing health care. Even before the COVID-19 pandemic, there were many barriers to accessing health care for transgender people, such as a shortage of skilled health professionals, resulting in very few transgender people receiving gender-specific surgery and hormone interventions, especially in low- and middle-income countries [4]. As a fringe group, inequalities faced by transgender people in politics and society, such as legislation based on binary gender norms, could increase the risk of disease and mortality during the COVID-19 pandemic [5]. To prevent overloading the health care system with COVID-19 cases, planned operations as well as examinations and therapies for non–life-threatening conditions have been postponed [5].

Due to pandemic containment measures, many patient appointments had to be cancelled or were switched to telephone or video counseling. However, the clinical care of patients had to continue. This made it even more difficult for transgender people to access hormone interventions and sex-affirming operations [6]. Because of the difficulties caused by COVID-19, it is likely that transgender people also face difficult situations in terms of their mental health. Recent studies found that difficulties in accessing hormones were associated with high levels of anxiety and depression, as the availability of future therapies was uncertain and transgender people still wanted treatment during the COVID-19 pandemic [7,8]. In particular, transgender patients are dependent on regular medical consultations. Follow-up checks are frequently carried out to monitor ongoing endocrine hormone therapy. Endocrine hormone therapy is essential for the physical transition; with its help, sufferers could have a normal life. New concepts and ideas have recently been introduced. The topic of digitalization was driven forward by the COVID-19 pandemic. The use of digital applications in everyday clinical practice is well established among cisgender people. While some medical disciplines have made more progress in the implementation and application of digital media, other disciplines remain largely untouched [9]. Digital media and applications can positively influence patient care and open up new treatment pathways. In general, many physicians believe that telemedicine has great potential for managing patient care. Digitalization affects 90% of the health care system and has already brought many changes for patients and doctors, which have decisively influenced the patient-doctor relationship [10]. Patients are willing to use mobile health technologies to improve their disease status and monitor symptoms and disease activity. The use of digital health applications has also increased in recent years [11]. Data from these applications have been obtained from patients and health care professionals. As with the general population, the internet and social media have been useful in reducing isolation during lockdowns and for this marginalized group (ie, transgender persons); they have also been important in helping this group maintain contact with health facilities through telemedicine services [12]. The perspective of transgender people and physicians is crucial for the successful development and implementation of telemedicine concepts for the management of transgender patient care [13]. The central question is whether and how adequate treatment can be delivered digitally in the future for this special group. This study explored the acceptance, use, and barriers of telemedicine in times of SARS-CoV-2 in transgender health care in Germany, as well as how the medical and mental health care of this special group of patients can be improved by the use of telemedicine applications. Changes in these aspects were observed, particularly during the COVID-19 pandemic.

## Methods

Surveys were conducted among gynecological endocrinologists (specialists and trainees) who provide gender-specific hormone treatments to transgender patients. In addition, we evaluated the perspectives of transgender patients undergoing gender-affirming hormone therapy (GAHT) regarding the use of digital health applications in the form of telemedicine in the times of COVID-19 during their GAHT. The responsible ethics committee of the University of Jena was informed of the study and did not object to it (registration No. 2019-1456-Bef). Web-based surveys were conducted by members of the Youth Working Group Forum of the German Society for Gynecology and Obstetrics (DGGG). In order to investigate the identified areas of interest, a panel of experts administered the study questionnaire during two separate online meetings based on individual literature searches, similar to the standard operating procedures drafted by the EULAR (European League Against Rheumatism) recommendation working group [14]. Four areas were investigated: (1) epidemiological data of respondents, (2) basic use of digital health applications, (3) knowledge and use of telemedicine, and (4) barriers and benefits of tele-endogynecology. The web-based study questionnaires were designed according to published guidelines for questionnaire research [15-17]. The choice of questions for the questionnaires was based on both comparable work and on the quality criteria for online questionnaires [18]. The two web-based surveys were created in SurveyMonkey (Momentive). The surveys were administered from November 1, 2020, to March 30, 2021. The data were collected anonymously. The methodology and results of the study were reported according to the Checklist for Reporting Results of Internet E-Surveys (CHERRIES) [19]. Content of the questionnaires was developed based on the published research results on digitalization among patients, a 23-part, self-managed online questionnaire. Members of the Youth Working Group Forum of the DGGG were asked to provide feedback on the format, completeness, clarity, and procedure of the validation process [16]. Both surveys were pilot-tested. The survey for physicians was tested on 10 physicians, and the patient survey was tested on 10 transgender patients; this was done to gauge the need to refine the wording and format and to check whether predefined response options were exhaustive. Minor revisions were made; accordingly, the questionnaires were modified. The surveys consisted of binominal questions, questions in categorical Likert-scale formats (6 levels), and open questions; the surveys were entitled “Telemedicine in the era of COVID-19 in gynecological endocrinology for the treatment of transgender patients.”

The main sections were as follows: (1) epidemiological data of respondents, (2) basic use of digital health applications, (3) knowledge and use of telemedicine, and (4) barriers and benefits of tele-endogynecology.

We aimed to shorten the interview duration using the surveys to a maximum of 15 minutes in order to keep the dropout rate as low as possible and to motivate the respondents, as much as possible, to answer all of the questions [20,21]. The physician questionnaire was distributed via email to the physicians. In an information letter, participants were informed that their data would be treated in a strictly confidential and anonymous manner and that they would be able to access the online questionnaire via a QR (Quick Response) code or survey link. All participants gave their consent digitally before the start of the study. To do this, they had to manually participate in the study by clicking the button “I agree to participate in the study.” If participants refused to participate in the study, their participation was terminated and was not evaluated. The physician survey was sent digitally to 2287 gynecological endocrinologists (specialists and trainees) in Germany who provide GAHTs to transgender patients. The contact details of potential participants in Germany were provided by the Association of Statutory Health Insurance Physicians [22] and are available to the public. In 2020, a total of 2942 consultations were conducted during the special consultation hours at the University Women’s Hospital Jena, and a total of 421 patients undergoing GAHT were treated by four gynecological endocrinologists. GAHTs were administered to female-to-male and male-to-female patients. All of these patients were contacted by postal letter. In an information letter, participants were informed that their data would be treated in a strictly confidential and anonymous manner, and that they would be able to access the online questionnaire via a QR code or survey link.

Exclusion criteria included the following: patients under 18 years of age, patients not currently undergoing GAHT, physicians without the medical designation of gynecological endocrinology, physicians not performing GAHT, and digital refusal to participate in the study.

The results were analyzed using SurveyMonkey and SPSS Statistics for Windows (version 27.0; IBM Corp). Descriptive statistics included quantities, percentages, median scores, and ranges for ordinal variables. The chi-square test was applied for the analyses of influencing parameters. *P* values of less than .05 were considered significant.

## Results

### Overview

From November 2020 to March 2021, a cross-sectional, self-administered, web-based survey regarding the acceptance, use, and barriers of telemedicine in times of SARS-CoV-2 in transgender health care in Germany was completed by gynecological endocrinologists and transgender patients in Germany. Of the 2287 physician questionnaires that were sent out, 286 (12.5%) were returned. Of the 286 responses, 84 (29.4%) were excluded from the analysis because fewer than half of the questions were answered. The final response rate for physicians was 8.8% (202/2287). In the period from November 2020 to March 2021, 333 out of 421 (79.1%) transgender patients participated in the study. Of the 333 responses, 64 (19.2%) were excluded from the analysis because fewer than half of the questions were answered. The final response rate for patients was 63.9% (269/421).

### Epidemiological Data of Respondents

A total of 202 gynecological endocrinologists and 269 patients completed the surveys. Most patients (n=115, 42.8%) were between 21 and 30 years old. The majority (n=187, 69.5%) of the participating patients had been undergoing GAHT for more than 24 months. Most of the GAHTs among the study participants were being carried out in the context of the transformation from female to male (n=148, 55.0%).

Of the 202 physician respondents, almost one-third were between 41 and 50 years old (n=69, 34.2%) and most of them were specialists in the field of GAHT (n=175, 86.6%). One-quarter of them were still in training to become gynecological endocrinologists (n=51, 25.2%) and around two-fifths were between the ages of 21 and 30 years (n=32, 15.8%). The smallest proportion of these respondents were over 60 years of age (n=25, 12.4%). Almost all of the physicians were women (n=148, 73.3%). A total of 44.1% (n=89) of the physicians worked in a private practice, 32.2% (n=65) were clinicians in a university hospital, and about one-quarter worked in a nonuniversity hospital (n=48, 23.8%). Details of the participants are given in Multimedia Appendix 1.

### Basic Use of Digital Health Applications

Out of 269 patients, 82.5% (n=222) reported using apps several times a day on a smartphone, 9.7% (n=26) used apps once daily, and 6.2% (n=17) used apps once weekly. Only 1.5% (n=4) of the patients stated that they never used apps. A total of 91.1% (n=245) of the patients were able to use digital health applications. In addition, 79.9% (n=215) said that the use of digital health applications can have a positive impact on their disease treatment, while 20.1% (n=54) disagreed. The higher the age of the patients, the lower their overall app usage (*P*<.001) and the lower their confidence in using apps (*P*<.001), adjusted for gender and time of treatment. All physicians were able to use digital health applications. A total of 66.8% (n=135) of the gynecological endocrinologists described the use of digital health applications for managing a patient’s disease as useful, while only 3.0% (n=6) disagreed. No significant difference in gender, age, degree of training, and workplace was noted. Due to the COVID-19 pandemic, the attitude toward digital health applications changed positively in 54.3% of patients (n=146) and 40.1% of physicians (n=81). A total of 88.8% of the patients (n=239) and 64.4% of the gynecological endocrinologists (n=130) reported using digital health applications more regularly (Multimedia Appendix 2).

**Figure 1:**
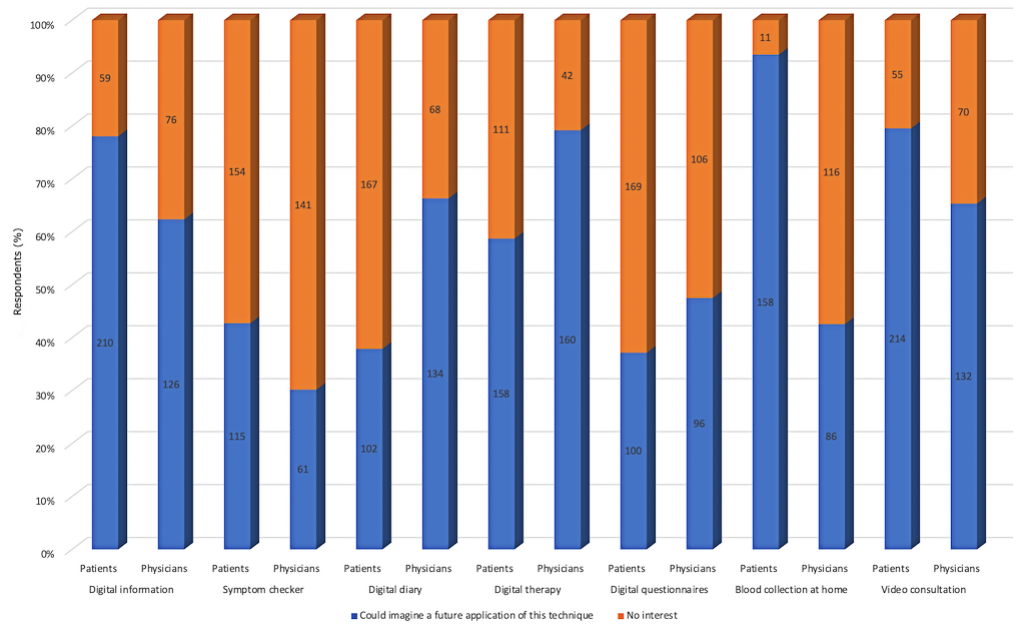
Details of the participants in the study. Numbers of respondents are reported on the plot bars.

**Figure 2:**
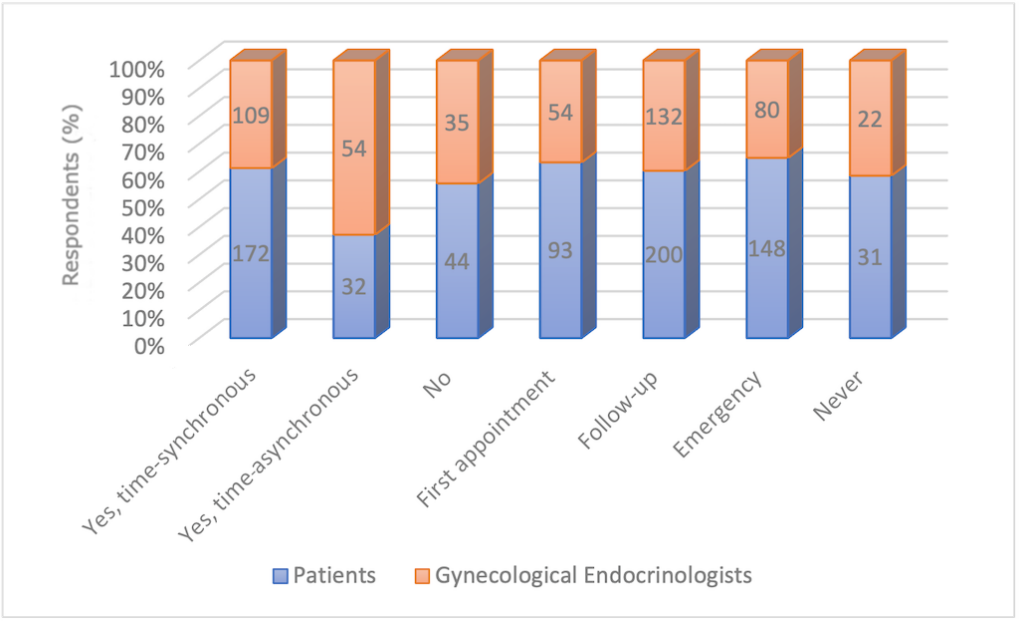
Respondents' attitudes to video consultation hours. Numbers of respondents are reported on the plot bars.

At the time of the survey, patients were most likely to use video consultations (n=214, 79.6%), informative digital health applications (n=210, 78.1%), and symptom checkers (n=115, 42.8%). Therapy-based digital health applications and self-drawn blood samples with digital access to the results showed different levels of acceptance: 58.7% of patients (n=158) said they had no interest and 41.3% (n=111) could imagine a future application of these techniques, respectively. Physicians were most likely to use therapy-based digital health applications (n=160, 79.2%), digital diaries (n=134, 66.3%), and video consultations (n=132, 65.3%). Self-drawn blood samples with digital access to the results showed different levels of acceptance: 57.4% of physicians (n=116) said they had no interest and 42.6% (n=86) could imagine a future application of this technique. The majority of gynecological endocrinologists rejected the use of symptom checkers (n=61, 30.2%). Details of the participants are given in Figure 1. Patients were most likely to say that video consultations for aftercare (n=200, 74.3%) and emergency appointments (n=148, 55.0%) were possible. A total of 63.9% (n=172) of patients said that time-synchronous digital consultation could complement physical appointments. In addition, 75.5% (n=203) of patients and 64.4% (n=130) of gynecological endocrinologists indicated that they should cancel an appointment on-site if the patient’s disease is stable and if he or she can indicate his or her well-being using a digital health application (Figure 2).

### Telemedicine From a Medical Point of View: Knowledge and Use

A total of 71.3% (n=144) of the 202 physicians rated their knowledge of telemedicine as 4 (unsatisfactory), 5 (bad), or 6 (very poor). The minority (n=58, 28.7%) rated their knowledge of telemedicine as 1 (very good), 2 (good), or 3 (satisfactory). At the time of the survey, the majority (n=175, 86.6%) of the physicians did not use telemedicine, but 69.3% (n=140) said they would like to use it. A total of 89.1% (n=180) of the physicians pointed out that they do not use telemedicine due to barriers. According to the respondents, the main obstacles to the introduction of telemedicine were the purchase of technological equipment (n=132, 65.3%), administration (n=124, 61.4%), poor reimbursement (n=106, 52.5%), lack of data security (n=92, 45.5%), less participation by colleagues (n=67, 33.2%), technical comprehension of patients (n=55, 27.2%), and poor internet connection (n=52, 25.7%) (Multimedia Appendix 3).

### Tele-endogynecology in Transgender Patient Care Management: Barriers and Benefits

Out of 202 physician respondents, 74.8% (n=151) considered telemedicine to be useful in gynecological endocrinology in transgender patient care management. When asked which interactions should occur within telemedicine, 82.2% (n=166) answered doctor-doctor interactions, 66.8% (n=135) answered doctor-patient interactions, and 35.6% (n=72) answered physician-assistant interactions (multiple answers were possible). The preferred therapeutic phases for the use of telemedicine in the treatment of transgender patients were the follow-up phase (n=150, 74.3%), first contact (n=68, 33.7%), and preventive examinations (n=39, 19.3%). Participants were asked to provide specific digital tools that could support endocrinological care for transgender patients undergoing GAHT. The most frequently selected topics were teleconsulting (n=128, 63.4%), video consultations (n=90, 44.6%), and tele-diagnostics (n=75, 37.1%). This was followed by online appointments (n=64, 31.7%), e-learning (n=45, 22.3%), patient apps (n=37, 18.3%), digital screening (n=35, 17.3%), portable devices (n=22, 10.9%), and other instruments (n=9, 4.5%) (Multimedia Appendix 4).

## Discussion

### Principal Findings

This study was the largest nationwide survey on the use of telemedicine in the field of gynecological endocrinology for the promotion and implementation of tele-endogynecology for the treatment of transgender patients in Germany. For this purpose, transgender patients undergoing GAHT and gynecological endocrinologists performing GAHT were interviewed. The two surveys evaluated the perspectives of patients and gynecological endocrinologists on the acceptance of, attitudes toward, and possible barriers to the use of telemedicine applications during GAHT and the impact of the COVID-19 pandemic on their use. The survey contained the following main topics: (1) epidemiological data of respondents, (2) basic use of digital health applications, (3) knowledge and use of telemedicine, and (4) barriers and benefits of tele-endogynecology.

In line with previous surveys [23,24], the respondents reported that they regularly used mobile apps on their smartphones and believed that they would be able to follow digital health advice. Other general studies on the use of digital media showed that with age, interest in digital health applications decreases among patients and physicians [25,26]. The patients in this study were very young compared to other studies. The age of the interviewed physicians compared to that of the patients was twice as high and ranged from 41 to 50 years. Comparing the user behavior of the young patient group with the group of physicians in this study, there was no significant difference in the basic attitude toward the acceptance and use of telemedicine applications. In this survey, transgender patients and gynecological endocrinologists reported a general positive attitude toward telemedicine and at least regular use of telemedicine applications. This may be the reason that transgender patients and gynecological endocrinologists reported a general positive attitude toward telemedicine. Both believed that the use of telemedicine in the form of digital health applications (eg, medical apps, video consultations, and online pharmacies) can improve disease management. This study’s findings about the fundamental benefits of using telemedicine for treatment in disease management are in line with previous work. For example, Enam et al found that the use of telemedicine can be evidence based if appropriate media are used; the data from Enam et al are from the time before the COVID-19 pandemic [27].

Respondents to our study indicated that the COVID-19 pandemic had a positive impact on attitudes toward telemedicine applications in the form of digital health applications. Not only was attitude toward telemedicine applications influenced by the COVID-19 pandemic, but user behavior was as well. More than three-quarters of the respondents said that the COVID-19 pandemic had increased their personal use of digital health applications. Transgender patients reported a higher use of these applications than physicians. As with the general population, during the lockdown, the internet and social media were useful in reducing isolation among transgender people and were also relevant for keeping in touch with associations and health care facilities with the support of telemedicine services [28]. Physicians’ use behavior had increased as a result of the COVID-19 pandemic; they were using digital health applications more often than before the pandemic. This seems to be a general development in the medical field, as shown by other studies [29]. The general increase in interest and acceptance of telemedicine among patients and physicians may provide the basis for longer-term use and increasing development of the use of telemedicine in the treatment of diseases, regardless of the disease or group of persons. This can be underlined by the findings of this study, which also showed that the patient population of this study is a special group of patients.

In their study, Winter et al [30] described the difficult access that transgender patients have to general health care facilities, the associated hurdles and barriers, and the mental health and social consequences they faced compared to the general population before the COVID-19 pandemic. The COVID-19 pandemic has particularly exacerbated the situation for this particular societal group. In Italy and a number of other countries, access to health care has been difficult or impossible for transgender people. This has obstructed the beginning or continuation of hormonal and psychic treatment among this group. In addition, planned gender-equalizing operations have been postponed. These obstacles have led to several problems for transgender patients. There has been an additional mental strain, and the positive effects of medical and surgical treatments on their well-being has been absent. Stressors have increasingly been directly and indirectly triggered, such as discrimination at work, social inequalities, and a deterioration in health care for this particular patient group [31]. This was investigated in a study by van der Miesen et al, in which they concluded that transgender patients are disadvantaged and often indeterminate; they determined that some organizational aspects should also be considered, since inequalities and marginalization of transgender subjects potentially increase the risk of morbidity and mortality [7]. This is where telemedicine can be used to limit such consequences and problems.

A study by Gava et al investigated endocrinological care of transgender patients during the COVID-19 pandemic [31]. They investigated the use of telemedicine in transgender patients as part of hormone treatment. Between May and June 2020, they conducted an anonymous web-based survey of transgender people in Italy. Among the 108 respondents, who had a mean age of 34.3 (SD 11.7) years, 73.1% were transgender men and 26.9% were transgender women. A total of 88.9% of respondents were undergoing GAHT. A total of 1 in 4 patients experienced a moderate to severe impact from the pandemic event. The availability of tele-endocrinological visits was associated with improved mental health scores. The survey suggested that there was a positive effect from telemedicine, as the availability of tele-endocrinological consultations may have relieved the distress caused by the COVID-19 pandemic by offering the opportunity to avoid halting GAHT. The age of the transgender patients and the proportion of female-to-male transgender patients in that study were almost identical to our study. We also investigated the use of telemedicine in transgender patients receiving hormone therapy in times of COVID-19. In the study by Gava et al, only transgender patients were interviewed, while the statements and attitudes of medical staff were completely lacking. Other published studies on transgender health and telemedicine dealt exclusively with the attitudes of the affected people themselves [12].

There is a complete lack of knowledge about the attitudes toward, and opinions on, telemedicine by the medical staff who would administer the therapy and, thus, be responsible for its administration. It is precisely these attitudes toward acceptance and application and barriers to telemedicine in the treatment of transgender people that are important, as they can fuel the basis for enabling change and progress of telemedicine when the individual backgrounds are known by the doctors who perform the treatment. With our study, we were able to identify initial insights into the relevance, application, and potential barriers to the use of telemedicine by physicians treating transgender people and performing medical treatments in the form of hormone treatments. All the physicians in our studies reported being able to use telemedicine applications, regardless of age and gender, which is the first prerequisite for the implementation of these novel applications. The interviewed physicians in this study came from the field of endocrinological gynecology, and our survey reflected only their opinions. The survey was aimed at gynecological endocrinologists from all over Germany, especially doctors from Thuringia and Bavaria, who participated in the recruitment strategy. A self-selection bias and a nonresponse bias in this study were possible because the survey was probably answered predominantly by doctors and patients interested in telemedicine. The survey was conducted in the time of COVID-19, and prepandemic data are pending in this area, so further research on the development of the acceptance of telemedicine applications in general and in relation to tele-endogynecology is urgently needed. An online survey was deliberately used to increase the response rate, since respondents could complete it quickly, regardless of place and time, and to achieve a reduced effort for data management. However, it is expected that this online survey will lead to a positive bias toward users of telemedicine. To answer the questionnaire, knowledge of the field of telemedicine was required (eg, preferences for specific tools were requested). Given the limited knowledge of doctors in the field of telemedicine, distortions were likely. In addition, we expect there to be rapid technological developments in the field of telemedicine, so the predefined response categories may not have been exhaustive enough.

### Perspectives of Telemedicine in Transgender Health Care Management

COVID-19 has increased the importance of contactless approaches to medical care. Already in 2020, when we conducted the survey, transgender patients and gynecological endocrinologists were willing to use tele-endogynecology. It is assumed that as a result of the pandemic there has been an increase in the willingness to speed up the use of telemedicine as part of social action and new standards in health care [23]. However, the maximum potential of telemedicine has not been fully achieved. Further research on implementation is urgently needed. This includes large-scale randomized controlled studies on the effects and health effects, risks and incidents, and specific interventions. Since our results showed that there was no “one-size-fits-all” solution in the field of telemedicine, the perspectives and preferences of physicians, patients, and others telemedicine users in tele-endogynecology are indispensable. This can create the basis for individual patient- and physician-adapted telemedicine options and triage mechanisms to select patients for digital or analog consultation, as appropriate [32,33]. As doctors reported regarding the barriers to the use of telemedicine, it seems that the structural framework for the effective implementation of tele-endogynecology is not yet in place. The use of telemedicine by the doctors interviewed was hindered by considerable administrative burdens and inadequate reimbursement structures. The biggest obstacle, however, was the limited knowledge of physicians about the use of telemedicine, which is why it is necessary to provide early information on telemedicine in the introduction of low-threshold training courses.

### Conclusions

Our study showed that gynecological endocrinologists and transgender patients support the implementation of tele-endogynecology, and two-thirds of those want telemedicine incorporated into their clinical routine. The medical professionals expressed an even greater willingness to use telemedicine. Respondents welcomed a variety of telemedicine approaches. However, at the time of the survey, only a minority of the interviewed physicians used telemedicine in their clinical routine. In addition, most physicians considered their knowledge of telemedicine to be rather poor. The provision of high-quality telemedical care requires additional research, a reduction in existing obstacles, and training for professionals and generalists. Transgender patients are very open to treatment with telemedicine applications. The foundations have been laid, and the concepts in this area have great potential for the future and should be developed.

## Appendix

Multimedia Appendix 1 Details of the participants.

Multimedia Appendix 2 Usage of digital health applications before and after COVID-19 pandemic.

Multimedia Appendix 3 Knowledge and use of telemedicine.

Multimedia Appendix 4 Implementation of telemedicine in transgender health care management.

## Abbreviations

CHERRIES: Checklist for Reporting Results of Internet E-Surveys
DGGG: Deutschen Gesellschaft für Gynäkologie und Geburtshilfe (German Society for Gynecology and Obstetrics)
EULAR: European League Against Rheumatism
GAHT: gender-affirming hormone therapy
QR: Quick Response
