# Supplementary material for: Acceptance, Use, and Barriers of Telemedicine in Transgender Health Care in Times of SARS-CoV-2: Nationwide Cross-sectional Survey
Source: JMIR Public Health Surveill. 2021 Dec 3;7(12):e30278. doi: 10.2196/30278 (PMC8647970; doi:10.2196/30278)
Supplement: Multimedia Appendix 1 [file publichealth_v7i12e30278_app1.docx]

**Multimedia Appendix 1.** Details of the participants.

| gynaecological endocrinologists, n=202 (100%) | Patients n= 269 (100%) |
| --- | --- |
| Women 148 (73) |  |
| Age (years) |  |
| 21-30 32 (16) | 115 (43) |
| 31-40 42 (21) | 86 (32) |
| 41-50 69 (34) | 35 (13) |
| 51-60 34 (17) | 22 (8) |
| >60 25 (12) | 11 (4) |
| Consultant 151 (75) | Female-to-male 148 (55) |
| Resident 51 (25) | Male-to-female 121 (45) |
| Working place | Treatment time |
| Private practice 89 (44) | > 24 month 187 (70) |
| University hospital 65 (32) | < 24 month 82 (30) |
| Non-university hospital 48 (24) |  |
