# Supplementary material for: Acceptance, Use, and Barriers of Telemedicine in Transgender Health Care in Times of SARS-CoV-2: Nationwide Cross-sectional Survey
Source: JMIR Public Health Surveill. 2021 Dec 3;7(12):e30278. doi: 10.2196/30278 (PMC8647970; doi:10.2196/30278)
Supplement: Multimedia Appendix 2 [file publichealth_v7i12e30278_app2.docx]

**Multimedia Appendix 2**. Usage of digital health applications before and after COVID-19 pandemic.

| **Characteristics** | **Patients n=269 (100%)** | **Gynaecological endocrinologists n=202 (100%)** |
| --- | --- | --- |
| **I believe using digital health applications (eg, medical apps, video consultation and online pharmacies) is useful for managing (my) disease, n (%)** | | |
| Strongly disagree | 19 (7.1) | 0 (0) |
| Disagree | 33 (12.1) | 6 (2.8) |
| Neutral | 2 (0.8) | 61 (30.3) |
| Agree | 165 (61.2) | 85 (42.2) |
| Strongly agree | 51 (18.8) | 50 (24.7) |
|  |  |  |
| **Has your attitude towards digital health Apps changed due to the COVID-19 pandemic?, n (%)** | | |
| It changed positively | 146 (54.4) | 81 (40.3) |
| It changed negatively | 23 (8.5) | 48 (23.5) |
| It has been unaffected | 100 (37.1) | 73 (36.2) |
|  |  |  |
| **Do you use digital health apps more regularly since the COVID-19 pandemic?, n (%)** | | |
| Yes | 239 (88.7) | 130 (64.6) |
| No | 30 (11.3) | 72 (35.4) |
|  |  |  |
| **I feel able to use digital health apps, n (%)** | | |
| Strongly disagree | 3 (1.2) | 0 (0) |
| Disagree | 5 (1.9) | 0 (0) |
| Neutral | 16 (5.9) | 0 (0) |
| Agree | 208 (77.2) | 151 (74.6) |
| Strongly agree | 37 (13.8) | 51 (25.4) |
