# Supplementary material for: Acceptance, Use, and Barriers of Telemedicine in Transgender Health Care in Times of SARS-CoV-2: Nationwide Cross-sectional Survey
Source: JMIR Public Health Surveill. 2021 Dec 3;7(12):e30278. doi: 10.2196/30278 (PMC8647970; doi:10.2196/30278)
Supplement: Multimedia Appendix 3 [file publichealth_v7i12e30278_app3.docx]

**Multimedia Appendix 3.** Knowledge and use of telemedicine.

| Question | responses gynecolocigal endocrinologists n (%) |
| --- | --- |
| How do you rate your own knowledge of telemedicine? |  |
| total | 202 (100) |
| 1 (very good) |  |
| 2 (good) |  |
| 3 (satisfactory) |  |
| 4 (unsatisfactory) |  |
| 5 (poor) |  |
| 6 (very poor) |  |
|  |  |
| Do you use telemedicne? |  |
| total | 202 (100) |
| yes | 27 (13.4) |
| no | 175 (86.6) |
|  |  |
| Would you like to use telemedicine? |  |
| total | 202 (100) |
| yes | 140 (69.3) |
| No | 62 (30.7) |
|  |  |
| Does anything prevent you from using telemedicine? |  |
| total | 202 (100) |
| Yes | 180 (89.3) |
| no | 22 (10.7) |
|  |  |
| What prevents you from using telemedicine?  (multiple selections possible) |  |
| total | 202 (100) |
| Purchase of technology equipment | 132 (65.3) |
| Administration | 124 (61.2) |
| Poor reimbursement | 106 (52.4) |
| Data security | 92 (45.6) |
| Lack of participation by colleagues | 67 (33.2) |
| Technical comprehension of patients | 55 (27.2) |
| Poor internet connection | 52 (25.7) |
