# Supplementary material for: Acceptance, Use, and Barriers of Telemedicine in Transgender Health Care in Times of SARS-CoV-2: Nationwide Cross-sectional Survey
Source: JMIR Public Health Surveill. 2021 Dec 3;7(12):e30278. doi: 10.2196/30278 (PMC8647970; doi:10.2196/30278)
Supplement: Multimedia Appendix 4 [file publichealth_v7i12e30278_app4.docx]

**Multimedia Appendix 4.** Implementation of telemedicine in transgender health care management.

| Question | responses gynecolocigal endocrinologists n (%) |
| --- | --- |
| Is telemedicine usable in gynecological endocrinology? |  |
| total | 202 (100) |
| yes | 151 (74.8) |
| no | 51 (25.2) |
|  |  |
| Which parties should establish communication via telemedicine? (multiple selections possible) |  |
| Total | 202 (100) |
| Physician-physician | 166 (82.3) |
| Physician-patient | 135 (66.7) |
| Physician-assistant | 72 (35.7) |
| Other participants and combinations | 41 (20.3) |
| No communication | 18 (8.9) |
|  |  |
| At which stages can telemedicine support transident patient care? (multiple selections possible) |  |
| Total | 202 (100) |
| Screening | 39 (19.4) |
| Initial contact | 68 (33.8) |
| Follow-up | 150 (74.4) |
| Other stages | 23 (11.4) |
| At no stage | 15 (7.4) |
|  |  |
| Which tools could support transident patient care? (multiple selections possible) |  |
| total | 202 (100) |
| Telecounseling | 128 (63.2) |
| Telediagnostics | 75 (37.1) |
| Video consultations | 90 (44.7) |
| Online appointment assignments | 64 (31.9) |
| e-Learning | 45 (22.4) |
| Patient apps | 37 (18.2) |
| Digital screening | 35 (17.2) |
| Wearable devices | 22 (10.8) |
| Other tools | 9 (4.4) |
| No tools | 5 (2.5) |
